# Fit for Surgery—feasibility of short-course multimodal individualized prehabilitation in high-risk frail colon cancer patients prior to surgery

**DOI:** 10.1186/s40814-022-00967-8

**Published:** 2022-01-21

**Authors:** R. D. Bojesen, L. B. Jørgensen, C. Grube, S. T. Skou, C. Johansen, S. O. Dalton, I. Gögenur

**Affiliations:** 1 Department of Surgery, Slagelse Hospital, Fælledvej 11, 4200 Slagelse, Denmark; 2 Center for Surgical Science, Zealand University Hospital, Køge, Denmark; 3 Department of Rheumatology, Section for Physio- and occupational Therapy, Zealand University Hospital, Roskilde, Denmark; 4 Department of Physiotherapy and Occupational Therapy, Næstved, Slagelse, Ringsted Hospital, Slagelse, Denmark; 5 Department of Sports Science and Clinical Biomechanics, University of Southern Denmark, Odense, Denmark; 6 Survivorship & Inequality in Cancer, Danish Cancer Society Research Center, Danish Cancer Society, Copenhagen, Denmark; 7 Psychological Aspects of Cancer, Danish Cancer Society Research Center, Danish Cancer Society, Copenhagen, Denmark; 8 Late Effect Research Unit CASTLE, Finsen Center, Rigshospitalet, Copenhagen, Denmark; 9 Department of Oncology & Palliative Care, Zealand University Hospital, Næstved, Denmark; 10 Department of Surgery, Zealand University Hospital, Køge, Denmark

**Keywords:** Prehabilitation, Colorectal cancer, Frail, High-intensity training, Elderly

## Abstract

**Background:**

Prehabilitation is a promising modality for improving patient-related outcomes after major surgery; however, very little research has been done for those who may need it the most: the elderly and the frail. This study aimed to investigate the feasibility of a short course multimodal prehabilitation prior to primary surgery in high-risk, frail patients with colorectal cancer and WHO performance status I and II.

**Methods:**

The study was conducted as a single-center, prospective one-arm feasibility study of eight patients with colon cancer between October 4, 2018, and January 14, 2019. The intervention consisted of a physical training program tailored to the patients with both high-intensity interval training and resistance training three times a week in sessions of approximately 1 h in length, for a duration of at least 4 weeks, nutritional support with protein and vitamins, a consultation with a dietician, and medical optimization prior to surgery. Feasibility was evaluated regarding recruitment, retention, compliance and adherence, acceptability, and safety. Retention was evaluated as the number of patients that completed the intervention, with a feasibility goal of 75% completing the intervention. Compliance with the high-intensity training was evaluated as the number of sessions in which the patient achieved a minimum of 4 min > 90% of their maximum heart rate and adherence as the attended out of the offered training sessions.

**Results:**

During the study period, 64 patients were screened for eligibility, and out of nine eligible patients, eight patients were included and seven completed the intervention (mean age 80, range 66–88). Compliance to the high-intensity interval training using 90% of maximum heart rate as the monitor of intensity was difficult to measure in several patients; however, adherence to the training sessions was 87%. Compliance with nutritional support was 57%. Half the patients felt somewhat overwhelmed by the multiple appointments and six out of seven reported difficulties with the dosage of protein.

**Conclusions:**

This one-arm feasibility study indicates that multimodal prehabilitation including high-intensity interval training can be performed by patients with colorectal cancer and WHO performance status I and II.

**Trial registration:**

Clinicaltrials.gov: the study current feasibility study was conducted prior to the initiation of a full ongoing randomized trial registered by NCT04167436; date of registration: November 18, 2019. Retrospectively registered. No separate prospectively registration of the feasibility trial was conducted but outlined by the approved study protocol (Danish Scientific Ethical Committee SJ-607).

**Supplementary Information:**

The online version contains supplementary material available at 10.1186/s40814-022-00967-8.

## Key points

1. What uncertainties existed regarding the feasibility?
  A short course of individualized multimodal prehabilitation prior to surgery with high-intensity interval training, resistance training, dietary and nutritional support, and medical optimization has not previously been described in high-risk frail colon cancer patients. Thus, it was not clear if it was feasible in regards to recruitment, retention, compliance and adherence, acceptability, and safety within this population.
2. What are the key feasibility findings?
  Compliance and adherence to the high-intensity interval training were high. However, using 90% of the maximum heart rate as a monitor of intensity was problematic. Compliance with the nutritional supplements was low.
3. What are the implications of the feasibility findings for the design of the main study?
  The high-intensity interval training should be monitored primarily by wattage, rather than the 90% of maximum heart rate. The nutritional supplements should be changed from an individualized dosage to a fixed amount in order to reduce the complexity of the interventions.

## Background

In the last decades, improved perioperative treatments, such as minimally invasive surgery, enhanced recovery after surgery, and early rehabilitation have dramatically reduced the overall early mortality for colorectal cancer patients [1–3]. The primary beneficiary of these improvements has been the younger group and to a lesser extent the elderly, frail, and comorbid patients [4, 5]. These patients have a markedly increased risk of postoperative morbidity and mortality [5] and would be expected to have the most to gain from additional efforts to improve the perioperative period.

One effort of improvement which may benefit the elderly and frail is prehabilitation [6] consisting of physical exercise, nutritional support, and medical optimization prior to surgery [7]. Each of the components focuses on known risk factors of poor postoperative outcomes [8–12], but the interventions have not been sufficient on their own to show a reduction in complications [13]. However, recent meta-analyses have shown that combining the interventions into multimodal prehabilitation may reduce the risk of postoperative complications and increase physical fitness after surgery [14–16]. Several concurrent studies are investigating prehabilitation, but most studies and described pilot studies included primarily younger and healthy individuals [17]. Both physical exercise, nutritional support, and medical optimization have each been shown to be feasible prior to surgery [9, 18, 19]; however, the feasibility of combining these often demanding interventions within an elderly and frail population is not clear.

One of the best predictors of postoperative morbidity and mortality after major abdominal surgery is reduced physical fitness measured by low oxygen uptake [20, 21], which potentially can be improved through training. Individualized high-intensity interval training (HIIT) has been shown to be a reliable way to improve physical fitness [22, 23]; however, it is not clear if a short course of HIIT can improve physical fitness in elderly and frail patients with colorectal cancer prior to surgery. Only one trial of multimodal prehabilitation focusing on elderly and frail patients has been published [24]. This trial used a combination of home-based and supervised training with moderate-intensity aerobic training and resistance training with elastic bands. Only adherence to the supervised training sessions was measured with a mean of 68% attendance. Compliance and acceptance with the training and nutrition were not described. In the literature, several aspects of the feasibility of multimodal prehabilitation in this population are not sufficiently described; thus, we planned to conduct a feasibility study in patients with higher WHO performance status and colorectal cancer before a randomized trial.

The primary aim of the study was to evaluate the feasibility of the protocoled individualized intervention regarding recruitment, retention, compliance and adherence, acceptability, and safety. Further, we wanted to describe the feasibility of measuring changes in physical fitness during the treatment course and report the challenges with a multimodal intervention within this population.

## Methods

### Design and setting

**Fig. 1:**
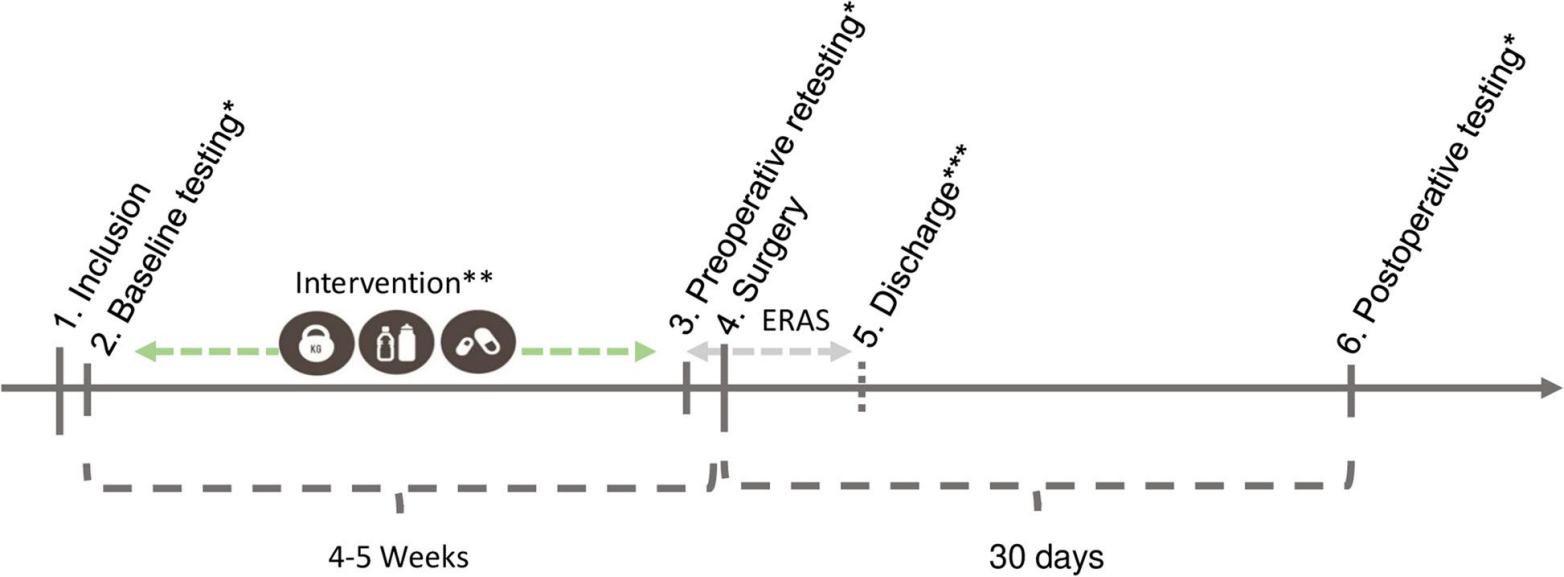
Outline of the course of testing and intervention. Single asterisk indicates the following: all testing consisted of baseline questionnaires (G8 and fried frailty), nutritional screening (PG-SGA) and anthropometric measurement, blood work, cardiopulmonary exercise test (CPET), handgrip strength, leg extension strength test, 6-min walk test, sit to stand test (30 s), and stair climb test (30 s), in that exact chronological order. Double asterisk indicates the following: the intervention consisted of individual training three times a week with a minimum of 10 sessions. Nutritional counseling within the first week of inclusion (1.5 h), 0.4 g/kg bodyweight protein supplement two times a day, and medical optimization. Medical optimization was performed on the same day of baseline testing. Triple asterisk indicates the following: discharge managed through standardized discharge criteria. Adherence to Enhanced Recovery After Surgery (ERAS) was recorded each day during admission

The study was conducted as a single-center, prospective feasibility study with eight patients undergoing multimodal prehabilitation prior to surgery for colorectal cancer. The study design was chosen with the aim of testing the feasibility of a complex multimodal intervention in patients with cancer and therefore not conducted with a control group. Patients were recruited from the Department of Surgery, Zealand University Hospital, Roskilde, Denmark, between October 4, 2018, and January 14, 2019. The intervention was planned in three standardized individualized intervention components: training intervention, diet and nutritional support, and medical optimization. All interventions were protocolled before the initiation of the study. A graphic description of the course of the intervention and testing can be seen in Fig. 1.

All patients received standard perioperative care, including a multi-disciplinary team conference, preoperative lung exercise education with positive expiratory pressure whistle, protein-enriched high-energy supplements 5 days prior to surgery, carbohydrate loading prior to surgery, and full enhanced recovery after surgery [25, 26]. The multi-disciplinary team conference consisted of a minimum of one senior radiologist, oncologist, and pathologist, besides the colorectal surgical team all with colorectal cancer as their area of expertise following the Danish national guidelines. Certified colorectal surgeons performed the surgeries.

### Participants eligibility

All patients with WHO performance status I or II, with planned surgery for colon or rectal cancer, without neoadjuvant radio- or chemotherapy were eligible for the study. Patients were excluded if they were planned for abdominoperineal resection, serum creatinine > 250 mmol/L, not able to understand or write Danish, had severe cognitive deficit (Mini-Mental State Examination < 1 1[27]), not able to perform exercise due to orthopedic impairments assessed by the principal investigator, known metastatic disease, or withdrew consent.

### The rationale for using WHO performance status as a screening tool

In Denmark, the government has introduced a cancer treatment guarantee, which means that patients are to undergo surgery within a maximum of 14 days from diagnosis to surgery. This study was approved to postpone the surgery for high-risk patients with potentially modifiable risk factors. Therefore, patients had to be assessed for eligibility shortly after referral to make sure that no patients had postponed surgery unless they were included in the study. For the majority of patients, WHO performance status is rated at referral, has good inter-observer reliability [28, 29], and is associated with an increased risk of postoperative morbidity and mortality [30], which makes it the most optimal available tool for screening. We chose to include patients with WHO performance status I and II, but not III or IV since these patients rarely are planned for surgery and were expected not to be able to perform the planned training intervention.

### Intervention

#### Training intervention

The training intervention consisted of high-intensity interval training (HIIT) and resistance training in supervised individual training sessions of approximately 1-h duration 3 times a week for at least 4 weeks. The HIIT was performed on an exercise bike and consisted of 4 min warm-up, followed by 4 bouts of 2 min at an intensity > 90% of the participants’ maximum heart. Between and after the bouts, low load intervals of a duration of 4 min were performed. Heart rate was continuously monitored by a chest-worn monitor (Polar A300®, Polar Electro, Finland). The threshold of 90% of the maximum heart rate was determined by a cardiopulmonary exercise test (CPET) in each participant. The participant was able to adjust the load, with the physiotherapist encouraging the participant to obtain the > 90% of maximum heart rate during the bouts. Self-exertion was rated by the participant using the Borg RPE 6-20 scale [31], after each bout and at the end of each HIIT training session describing the overall intensity of the complete session. The Borg RPE 6-20 measurements after each bout were used to guide intensity during the training, and the measurement after each training was used as a measure of compliance. After the HIIT session, resistance training was performed using machines (Technogym®, Italy) in the following order; chest press, lateral pulldown, and leg press. Three sets of 8–12 repetitions were performed in each machine. An 8-repetition maximum test (8-RM) [32] was used to calculate 1-RM. Resistance was set to progress throughout the intervention using the following model: week 1 (65% of 1-RM), week 2 (70% of 1-RM), and weeks 3, 4, and 5 (75% of 1-RM). All training was supervised by the same team of physiotherapists. Besides HIIT and resistance training, the patients were encouraged to perform light to medium aerobic exercise for at least 30 min a day at home. This was not supervised or registered.

#### Dietary and nutritional intervention

The nutritional intervention consisted of an addition of 0.4 g/kg bodyweight protein two times a day by TMP-90 Shake® (Friesland Campina, Netherlands) regardless of nutritional status or weight loss. One dose should be taken just after exercise or training and one before sleep. In addition, a multivitamin tablet (Apovit Multi®, Apovit, Denmark) with 100% of the daily recommended dosage, and a D vitamin with calcium tablet (38 μ + 400 Unikalk Mega®, Orkla Health A/S, Denmark) was taken daily. The dietary intervention consisted of an interview with a dietician within the first week after baseline testing. Estimation of current intake was done by a 24-h food recall, and daily dietary needs were conducted by estimation of base protein and total energy requirements by Harris-Benedict equation [33], with an added factor of 1.3–1.5, depending on the individual physical activity level. Patients were then advised how to change their diet to meet the excess demand for energy and protein, and if necessary instructed in using additional protein and energy drinks.

#### Medical optimization

The principal investigator performed medical optimization as part of the baseline testing and interview. Expanded routine blood work was performed including anemia parameters, hemoglobin A1c, cholesterol, vitamin status, minerals, white blood cell count, liver, and kidney parameters. If any unknown or poorly regulated disease was suspected, the patient was either referred to a specialist or adjusted in the medication, depending on the issue. Anemia needing correction was defined as ≤ 11.3 g/dL for both men and women, and patients were referred to intravenous administered iron(III) isomaltoside (Monofer® Pharmacosmos A/S, Denmark) at the earliest convenience. Medical history was assessed, and current medication was inspected for possible seponation or dose reduction. Patients with a high-risk intake of alcohol or smokers were encouraged to quit and if interested referred to the in-hospital alcohol and tobacco cessation course.

The complete intervention described in protocols translated into English can be found in the supplementary material (Appendix 1, 2 and 3). Full reporting of the training intervention in regards to Consensus on Exercise Reporting Template (CERT) guidelines [34] for reporting exercise interventions can be found in the supplementary material (Appendix 4).

### Testing

Testing was performed at baseline, the day prior to surgery, and 4 weeks after surgery. Testing included cardiopulmonary exercise test (CPET) and five physical function tests: handgrip strength, isometric leg extension strength test [35], 6-min walk test, 30 s sit to stand test [36], and 30 s stair climb test, in this exact order. The principal investigator performed all baseline testing. Pre- and postoperative testing (a day prior to surgery and 4 weeks after surgery) was performed by a physiotherapist specially trained to perform the testing procedures. CPET was performed on Jaeger® Vyntus® CPX (CareFusion, San Diego, USA) by steep ramp until exhaustion, with an expected testing time of 8–12 min. Handgrip strength was measured using a hand dynamometer (Jamar Smart®, Patterson Medical, Saint Paul, MN, USA) with three measurements on each hand, starting with the left hand. Isometric leg extension strength test was performed with a Lafayette Manual Muscle Tester (model LIC.01165, Lafayette Instrument Company, Lafayette, IN, USA) with three measurements on each leg, starting with the left leg. A 6-min walk test was performed in an undisturbed hallway on a 20-m course. Sit to stand was performed as described by Jones et al. [37]. A stair climb test was performed in an undisturbed stairwell with 10 steps of 17 cm on each floor, with a maximum of twelve floors available. The number of steps achieved within 30 s was recorded. Test of strength for chest press, lateral pull-down, and leg press was conducted as an 8-RM test and a 1-RM calculated from Brzycki’s formula [38]. Anthropometric measurements including skin fold, circumference, weight, and height were performed together with Patient-Generated Subjective Global Assessment (PG-SGA) [39] which was used for nutritional screening. Frailty was assessed by both the G8 score and Fried frailty [40] at baseline.

### Feasibility evaluation

#### Recruitment and retention

The feasibility of recruitment was evaluated as the percentage of eligible and included eligible patients that could perform the baseline tests. No predefined feasibility goal regarding recruitment was protocolled. Retention was evaluated as the percentage of included patients that concluded the intervention, with a predefined feasibility goal of 75% completing the intervention.

#### Compliance and adherence

The time spent above 90% of maximum heart rate during HIIT was of primary interest as a measurement of per-protocol compliance. Total time spent above 90% of maximum heart rate during HIIT was measured for each training session and each bout. We considered complete training as a minimum of 4 min of training above > 90% of maximum heart rate and with an overall Borg’s RPE > 16 of the training session and used this as a goal of compliance of the training intervention. Compliance with the nutritional support was predefined as ≥ 65% of both protein and vitamin ingestion. Patients received a fixed amount of protein supplement at baseline and the day prior to surgery the residual supplement was measured and usage was calculated as a percentage of the required intake. Adherence to the training intervention was measured by the percentage of completed training sessions. The predefined overall goal of adherence to the training was an attendance > 66% of maximum possible sessions. Adherence to the dietary and nutritional intervention was not measured.

#### Acceptability

Data on the acceptability of the interventions was obtained through note-taking during sessions and interviews with the participants at the preoperative assessment. No predefined measure of feasibility in regards to acceptability was protocolled.

#### Safety

The safety of the intervention was evaluated by registration of adverse events during the intervention and was evaluated by an external assessor through scrutiny of medical records. Muscle soreness was expected and all postoperative adverse events were not considered adverse events. No predefined measure of feasibility in regards to safety was protocolled but we aimed to describe all undesirable experiences occurring to the patients during the intervention no matter whether considered related to the intervention or not.

### Feasibility of outcome assessments

#### Baseline and treatment characteristics

Patient and disease-specific characteristics were collected at baseline. WHO performance status and ASA score were estimated by the surgeon at the first visit in the outpatient visit. Charlson comorbidity score was calculated including tumor and age [41]. Frailty was determined by a least one positive criteria on Fried frailty [40, 42] or by a Geriatric-8 (G8) score < 14 [43]. Perioperative treatment characteristics were collected 30 days postoperatively through scrutiny of medical records.

#### Clinical outcome measures

The primary clinical outcome measure of interest of test feasibility was changes in the maximum oxygen intake (VO_2_ peak) between baseline and preoperative assessment by CPET and the percentage of non-responders. VO_2_peak was estimated at the maximum oxygen uptake/min/kg body weight. Previous studies on repeated CPET´s have found a biological variation of VO_2_peak on 3.9% and an analytic variation of 2.2% [44]. Non-responders were defined as having an increase of less than 5% in the VO_2_ peak. Secondary clinical outcomes measures were as follows: change in maximum wattage during CPET, muscle strength, functional capacity, body weight, and albumin, postoperative length of stay, and complications within 30 days after surgery. Evaluation of postoperative complications was performed by an external assessor through medical records and graded by both the Clavien-Dindo classification [45] and the Comprehensive Complication Index [46]. Any readmission and Days at Home within 30 days after surgery (DAH-30) [47] were assessed through entries in medical records.

### Statistics and reporting

No statistical analyses were performed. Reporting was conducted in adherence to the CONSORT statement for feasibility and pilot studies [48], and the checklist can be found in the supplementary material. Due to the non-randomized design, several of the items are reported as “not applicable.” Complete reporting of the intervention in regards to CERT guidelines is presented in the supplementary material due to the complexity and multiple interventions within the study.

## Results

**Fig. 2:**
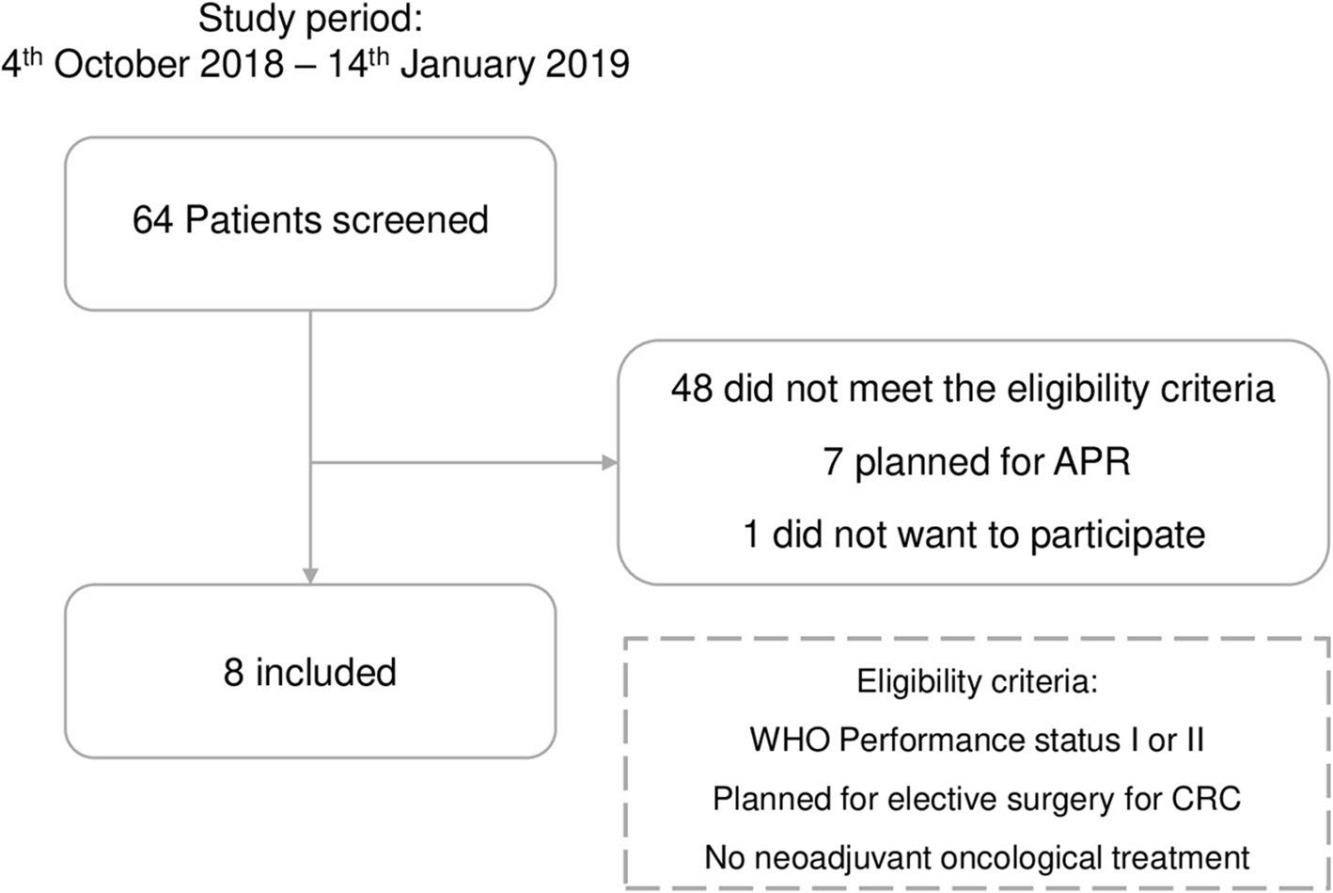
CONSORT diagram of the inclusion process. APR, abdominoperineal resection. CRC, colorectal cancer. WHO, World Health Organization

During the study period, 64 patients were screened for eligibility, and subsequently, 8 colon cancer patients were included in the study (Fig. 2). Subsequently, the diagnosis of one patient was revised confirming a benign pathology. However, this patient was treated following the oncological protocol and retained in the study. Table 1 shows that four men and four women were included with a mean age of 80 [range: 66–88]. At baseline, three patients had WHO performance status II, and five had performance status I. Six patients reported involuntarily weight loss during the last 6 months. Six patients had a G8 score < 14, and seven had at least one positive criteria in Fried frailty. Four patients had a hemoglobin level ≤ 11.3 g/dL.

**Table 1:** Patient characteristics at baseline

Patient	P1	P2	P3	P4	P5	P6	P7	P8
Age	66	86	86	78	88	80	78	79
Cancer	Colon	Colon	Colon	Colon	Colon	Colon	Colon	Colon
Gender	Female	Female	Female	Male	Male	Female	Male	Male
ASA	2	3	3	2	2	3	2	2
WHO PS	1	2	2	1	1	2	1	1
Charlson Comorbidity index	6	6	6	6	6	9	6	5
BMI (kg/m^2^)	29.1	24.6	22.6	38.5	21.5	27.8	19.5	25.9
Smoking status	Previous smoker	Non-smoker	Non-smoker	Non-smoker	Previous smoker	Previous smoker	Active smoker	Previous smoker
Alcohol consumption	1 U/week	0 U/week	0 U/week	5 U/week	10 U/week	0 U/week	21 U/week	14 U/week
> 5 medications	−	−	+	+	−	+	+	−
% weight loss during the last 6 months	0%	2.8%	9.1%	0%	8.6%	2.6%	9.0%	3.4%
Primary education	College level	Vocational trained	Vocational trained	Vocational trained	University	Primary education	College level	Vocational trained
T stage	3	2	2	2	3	2	4	2
N stage	2	0	0	0	0	0	1	1
G8 score	12	7	11	14.5	8	9	9	15
Fried frailty positive criteria	1	2	2	1	4	3	4	0
Hgb (g/dL)	8.06	10.8	12.25	13.05	12.57	11.28	9.83	11.44
Albumin (g/L)	35	38	38	36	34	30	32	33
CRP (mg/L)	6.7	< 2.9	< 2.9	< 2.9	6.3	17	2.9	23

*ASA* American Society of Anesthesiologists classification, *BMI* body mass index, *WHO PS* WHO performance status

### Feasibility evaluation

#### Recruitment and retention

Both patients with colonic and rectal cancers were eligible for enrolment; however, no patients with rectal cancers met the inclusion criteria within the study period. During the study period, nine eligible patients were approached for inclusion and only one patient declined to participate due to a scheduling conflict, leaving eight participating patients who were all able to perform the baseline test. Seven patients completed the intervention, while one patient developed an abscess in the tumor needing hospitalization and percutaneous drainage and could not complete the intervention. Further, three patients were unable to perform the four-week postoperative testing due to complications. One patient developed previously unknown ECG changes on the baseline CPET, which was consulted with a cardiologist, but without the need for change in medication or further diagnostics.

#### Compliance and adherence

**Fig. 3:**
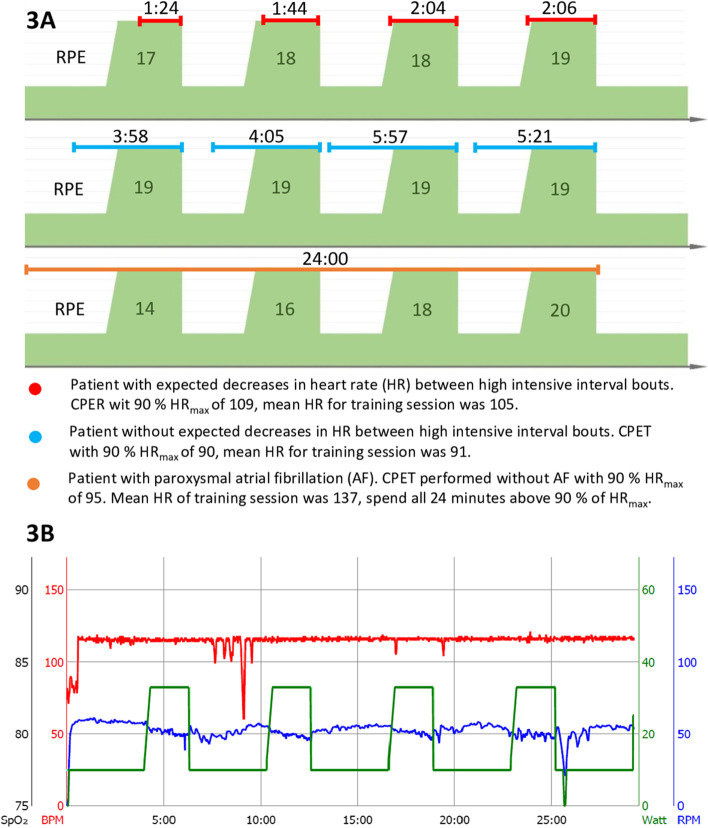
**a**, **b** Examples of different issues within training sessions based on maximum heart rate. **a** Examples of training sessions with continuous measurement of heart rate and Borg’s RPE for three different patients. Horizontal lines represent the time within the interval spent above 90% of maximum heart rate (HR) within each interval. Patient 1 (red) represents the expected course of the HR during a training session. Patient 2 (blue) shows a training session where the HR did not decrease between high-intensity intervals. Patient 3 (orange) shows a patient with known paroxysmal atrial fibrillation, which is suspected to have atrial fibrillation during the training. HR was above 100% of the maximum HR completely during the session. HR during the last 4 min of training was not registered. **b** Illustrative example of a high-intensity interval training bout of a frail patient with colonic cancer without an expected decrease in heart rate between high intensive interval bouts. Similar to patient two (blue) in Fig. 2 a. The example was produced on Lode Corival rehab ergometer bike (Lode B.V., Groningen NL) on a patient not included in the feasibility study, in order to show the missing decrease in HR in correlation to intervals on a similar patient. The measure of Watt (green line), revolutions per minute (RPM (blue line)), heart rate (beats per minute, BPM (red line)), by time in minutes (*x*-axis). Oxygen saturation (SpO_2_) was not measured. The resting pulse of 75, increased rapidly after the start of the bout, even on 30% of maximum wattage defined by CPET, and reached maximum pulse within the first minute of exercise. The pulse did not decrease in low-intensity phases. CPET, cardiopulmonary exercise test. RPE, Borg’s Rating of Perceived exertion (RPE) 6-20 scale

A total of 82 (87%) out of 94 possible training sessions were performed, but with great variability between patients (54 to 100%). The mean Borg’s scale estimation for training sessions was 17 (range: 15–18.5). In three patients, we were not able to get an accurate heart rate either due to severe scoliosis (*n* = 1) or atrial fibrillation (*n* = 2). For the monitored training sessions, 87% met the goal of compliance of 4 min > 90% heart rate. For three patients, we observed no drops in heart rate between bouts in the low load-interval phase; thus, 40% of training sessions were performed with more than 8 min of > 90% in heart rate during the high interval bouts (Fig. 3 a, b). Four patients reached the goal of the nutritional support of > 65% of ingested protein supplements. The main reason for not ingesting the protocolized protein intake was due to taste and texture and for one patient nausea and stomach aches. Four patients with anemia received intravenous iron with a single dose of iron(III) isomaltoside. No patients had changes in their medication as part of the medical optimization.

#### Acceptability

All eight patients expressed satisfaction with the intervention; however, four felt somewhat overburdened by the additional number of appointments and data reporting. Six patients reported difficulties with the weight-dependent dosages of protein supplements along with taste and texture. All patients reported some degree of empowerment through the intervention and appreciated the individually supervised training sessions. Several patients reported increased levels of physical exhaustion as a result of the training, however, without limiting them in their normal daily activities. The only patient, who smoked was not interested in smoking cessation. The same patient had an alcohol intake above recommendations but reduced intake to zero without the need for further counseling.

#### Safety

Two patients experienced a potential adverse event during the intervention, one had a urinary tract infection, and one developed an abscess in the tumor. Three patients reported a reduction in pre-existing pain and one reduced the need for pain medication. No adverse events were observed during the training and for the dietary and nutritional intervention, only the abovementioned patient with nausea and stomach ache was observed.

### Feasibility of clinical outcome assessments

**Fig. 4:**
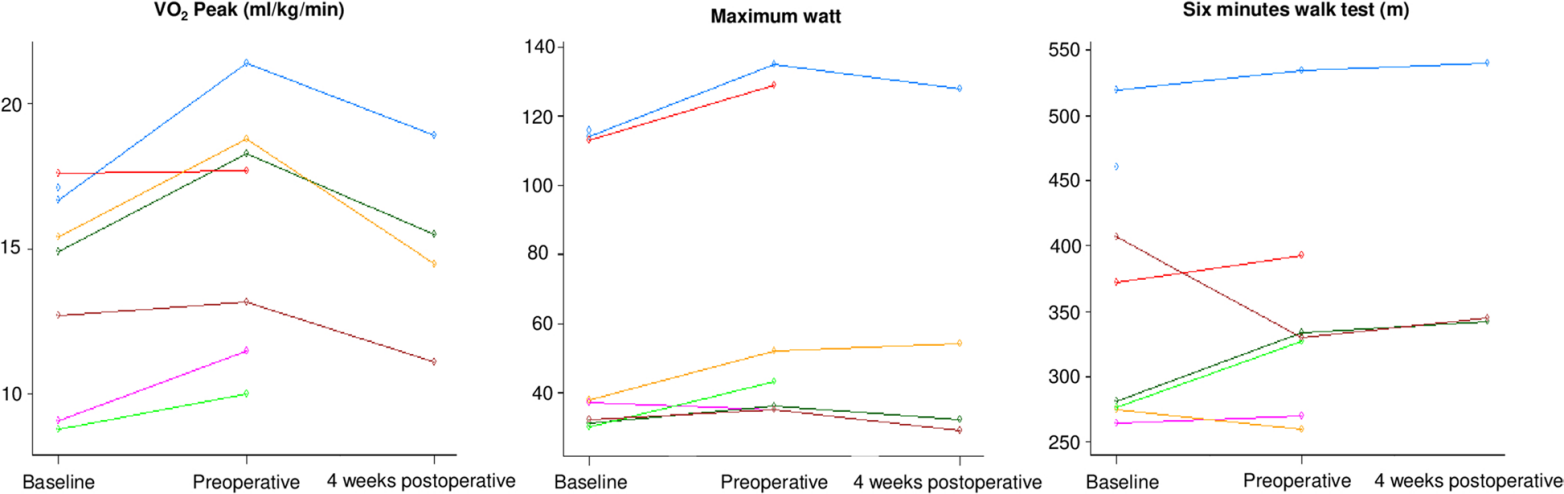
Changes in VO_2_ peak, workload, and 6-min walk test at baseline, preoperative, and 4 weeks after surgery for each patient

All seven patients that concluded the intervention performed both the baseline and preoperative CPET. The mean baseline VO_2_peak was 14 ml/min/kg [range: 8.8–17.6], with a mean maximum heart rate of 112 [range: 86–134]. The mean change in VO_2_ peak between baseline and preoperative testing was 17% [range: 0.6–28%], with two patients being non-responders regarding the VO_2_peak, but both these patients increased in maximum wattage (9% and 14%) (Fig. 4). Description of changes in physical fitness is shown in Table 2. Five patients had a self-reported weight loss the last six months before diagnosis, and all five increased their body weight, with a mean of 2.5 kg [range: 1.3–4 kg] during the intervention. None of the patients experienced weight loss during the intervention.

**Table 2:** Changes between baseline testing and prior to surgery, and perioperative outcomes

Patient	P1	P2	P3	P4	P5	P6	P7
Intravenous iron	+	+	0	0	0	+	+
Percentage of protein ingested	99%	17.5%	72%	73%	58%	74%	21%
**Change in physical capacity between baseline and prior to surgery**							
VO_2_ at AT (ml/kg/min)	3.7 (39%)	0.6 (9%)	3.7 (40%)	1.7 (15%)	− 4.2 (− 33%)	1.7 (29%)	− 1.8 (− 18%)
Handgrip strength (kg)	12.8 (55%)	0.3 (1.4%)	− 0.8 (− 8%)	− 6.7 (− 19%)	− 2.2 (− 8.2)	0.5 (2%)	− 13.5 (− 34%)
6-MWT (m)	15.3 (3%)	6 (2%)	53 (19%)	20.3 (5%)	− 15.2 (− 6%)	50.6 (18%)	− 77.3 (− 19%)
STS (repetitions)	− 1 (− 9%)	0 (0%)	1 (11%)	0 (0%)	0 (0%)	1 (11%)	− 1 (− 9%)
Stair Climb Test (number of stairs)	2 (4%)	− 4 (− 13%)	1 (3%)	2 (6%)	5 (22%)	5 (36%)	–
Chest press^a^ (kg)	6 (23%)	–	0 (0%)	11 (28%)	7 (100%)	2 (20%)	− 4 (− 24%)
Pull-down^a^ (kg)	7 (24%)	9 (25%)	1 (5%)	1 (2%)	8 (25%)	2 (8%)	− 7 (− 26%)
Leg press^a^ (kg)	58 (37%)	− 11 (− 18%)	2 (5%)	58 (38%)	14 (36%)	22 (34%)	− 25 (− 38%)
**Change in blood work between baseline and prior to surgery**							
Change in Hb (g/dl)	5.64 g/dL	0 g/dL	− 0.64 g/dL	− 0.64 g/dL	− 0.48 g/dL	1.93 g/dL	0.16 g/dL
Change in albumin (g/L)	3 g/L	1 g/L	0 g/L	− 2 g/L	2 g/L	8 g/L	− 1 g/L
Change in CRP (mg/L)	1.9 mg/L	0 mg/L	0 mg/L	0 mg/L	7.7 mg/L	0 mg/L	27.1 mg/L
**Perioperative and postoperative outcomes**							
Conversion of laparoscopy	−	−	−	+	−	−	−
LOS	2	3	1	4	2	26	6
Readmission	−	+	−	+	−	−	−
DAH-30	+	+	+	0	+	0	+
Stoma	−	+	−	−	−	+	−
Complication^b^	−	2	−	4a	−	4a	−
Day for full mobilization	1	1	0	2	1	?	3
Day for removal of urinary catheter	0	0	0	3	0	?	0
Day for starting oral nutrition	0	0	0	0	1	2	0

All measurements are given in absolute values between baseline and prior to surgery, with the change in percentage from baseline in parenthesis

*AT* anaerobic threshold, *DAH-30* Days at Home within 30 days after surgery, *Hb* hemoglobin, *LOS* length of stay, *STS* sit to stand in 30 s, *VO*_*2*_ oxygen uptake

^a^Indirect one-repetition maximum test calculated by Brzycki’s formula

^b^Highest complication graded by the Clavien-Dindo classification. Data from patient 8 is not presented since postoperative testing was not conducted in this patient due to an abscess in the tumor needing hospitalization and percutaneous drainage

Of the seven patients who completed the intervention, six had laparoscopic/robotic-assisted surgery, four of which had primary anastomosis. Conversion to laparotomy was performed in one patient, due to adherence of the tumor to the retroperitoneum. The mean surgical time was 195 min [range: 106–324 min]. The median length of hospital stay was 3 days [range: 1–26]. Two patients were subsequently readmitted within 30 days of discharge; one for a urinary tract infection and one for fascia dehiscence from an open laparotomy incision. Three patients developed a postoperative complication (one scored Clavien-Dindo 2 [Comprehensive Complication Index: 20.9], and two scored Clavien-Dindo 4a [Comprehensive Complication Index: 42.9 and 69.8]).

## Discussion

This feasibility study of multimodal prehabilitation, which demonstrated that frail high-risk colon cancer patients undergoing major abdominal surgery generally were interested in prehabilitation with a very low dropout rate, could comply and adhere to the training with a good acceptance rate. However, the compliance with nutritional support was low.

The study had a very high inclusion rate with eight patients included out of nine approached. This was surprising due to the complex and demanding study design including solely frail patients and postponing the surgery by approximately 2 weeks. We suspect that the included participants had a good understanding of the underlying hypothesis and general acceptance of physical training and nutritional supports as a means to improve their health status. This is supported by the high completion rates in which seven out of eight participants concluded the intervention with no voluntary discontinuation of the intervention and the high level of acceptance of the training intervention. However, in future clinical randomized trials, somewhat lower recruitment and completion rates should be expected.

The primary concern with the protocol was the use of heart rate to monitor the intensity of training. Several of the participants spent their complete training session above 90% of their maximum heart rate. From studies of HIIT in heart failure patients, heart rate seems to be a reliable monitor of intensity [49], but in our study population, we did not see the same cardiac adjustment of the load. Rather, the patients increased to their maximum heart rate rather rapidly and seemed to be capped there. Further, in three out of the seven patients, we were not able to reliably estimate their heart rate during the training sessions due to physical attributes mainly affecting the elderly population. One solution would be to use watt (SI unit: m^2^*kg*s^−3^) as the monitor of load, but this requires further baseline testing either as CPET or as a steep ramp test before planning the individual training plan. This would be preferable in the many elderly with paroxysmal atrial fibrillation in which the heart rate does not decrease between high-intensity intervals. On the other hand, fixed wattage, as seen in Fig. 3 b, does not take variability in physical performance between training sessions into account, which could potentially result in undertraining for some patients.

We did not have any follow-up or objective measurement of adherence to the dietician's advice, besides the increase in body weight and serum albumin. The compliance to the protein supplements was low with only four out of seven reaching the aim of > 65% ingested. This was particularly prominent in patients with higher body weight, illustrating the limitation of a weight-based dosage. For example, one of the patients was prescribed more than 100-g protein supplements a day. Furthermore, the weight-based dosage required weighing the supplements at home, rather than using a scoop which would be less cumbersome for the patients. This also showed the limitations of a multimodal individualized intervention in the outpatient setting. Each part of the prehabilitation intervention required a high degree of participation and resources from the participants, which we suspect that for some patients, surpassed their capacity. Thus, compliance varied considerably between participants especially in regards to the supplements. When planning future interventions this should be kept in mind and the complexity of the intervention should be reduced to a minimum.

Measuring the change in VO_2_ peak between baseline and preoperative testing was feasible and seemed reliable as an outcome assessment. The increase in VO_2_ peak was greater than previously described with HIIT in a healthy elderly population [50] but similar to what has been described in lung cancer patients [51, 52] and healthy colorectal cancer patients [53]. There is both a placebo and a learning effect when conducting repeated measurements of physical function; this includes CPET and the measurement of VO_2_max. It is not clear how much the increase in VO_2_peak is related to repeated testing in this population, and future studies should be careful when using the data in sample size calculations. Further, missing data of physical testing in the postoperative period are of concern, since it was only feasible in patients without complications and make any conclusion on maintenance in physical function 4 weeks after surgery difficult. The testers of the preoperative and 4-week postoperative tests were not blinded to the first CPET test results or the progression during the training period since data was used to estimate the load used for testing. Further, different testers performed the baseline-, the preoperative, and 4-week postoperative tests which potentially could lead to a systematic difference. This could be solved by a separated blinded testing team and with each test for each patient performed by the same tester.

A major strength was that training was supervised by the same team of physiotherapists and that all training was individually supervised and adapted which probably played an important role in the high compliance and retention. Further, the testing was standardized, performed in the same order for each participant, and each test. The high degree of adherence to enhanced recovery after surgery is another strength because the results of prehabilitation can then be treated as a therapeutic add-on to an optimal clinical setting. There were some potential adverse events and complications after surgery, as well as a very short length of hospital stay; however, further confirmatory trials are needed to ascertain these findings.

This one-arm feasibility study indicates that multimodal prehabilitation including HIIT, resistance training, protein supplementation, and medical optimization is feasible in elderly frail patients with colon cancer. Further research is necessary to make any clinical conclusions, especially randomized trials within elderly frail patients since they have the highest risk of not recovering after surgery and the most to gain from prehabilitation. Efforts should be made to develop and validate effective screening tools for high-risk patients who may benefit from prehabilitation efforts.

### Patient and public involvement

Prior to initiation of the study a patient panel of 10 colorectal cancer patients was asked to evaluate the proposed intervention. Nine out the ten found the duration and contents of the intervention acceptable and would participate in the study if given the opportunity. No major changes to the intervention were made to the intervention following the patient panel. Further, the patients included in the study were offered to participate in a semi-structured interview after completion of the intervention with two impartial representatives with the focus of evaluating the intervention and study design. The main concern with the intervention was logistical and did not alter the intervention.

## Supplementary Information

**Additional file 1.**

## Data Availability

The technical appendix is presented in the supplementary material. Raw data is presented in the paper. An anonymized version of the datasets used during the study is available from the corresponding author at a reasonable request.

## Abbreviations

ASA: American Society of Anesthesiologists
CERT: Consensus on Exercise Reporting Template
CPET: Cardiopulmonary exercise test
HIIT: High-intensity interval training
PG-SGA: Patient-Generated Subjective Global Assessment
VO_2_peak: Maximum oxygen uptake
WHO: World Health Organization
1-RM: One-repetition maximum
8-RM: Eight-repetition maximum
