# Supplementary material for: Fit for Surgery—feasibility of short-course multimodal individualized prehabilitation in high-risk frail colon cancer patients prior to surgery
Source: Pilot Feasibility Stud. 2022 Jan 21;8:11. doi: 10.1186/s40814-022-00967-8 (PMC8781359; doi:10.1186/s40814-022-00967-8)
Supplement: Supplementary file 1 — Additional file 1. [file 40814_2022_967_MOESM1_ESM.docx]

## Appendix 1: the training manual

**Translation:**

The training manual was translated into English from original Danish. The original protocol contains information about registration in electronic health records and other local information, which are omitted in the translated version. The original Danish protocol can be requested by contacting Rasmus Dahlin Bojesen [radb@regionsjaelland.dk](mailto:radb@regionsjaelland.dk) or [rasmus.bojesen@gmail.com](mailto:rasmus.bojesen@gmail.com).

**Fit For Surgery – Physiotherapists manual**

**Developed by**

Lars Bo Jørgensen and Søren Thorgaard Skou

**Field of application**

This training manual was developed for the project *‘Fit for Surgery‘* to describe the training of included participants.

**Overall conditions**

The training will be performed as supervised training by a physiotherapist. The training contains two elements: High-intensity interval training (HIIT) and resistance training in this chronological order. Data collection during training is performed during the training in the training log, and documentation (e.g. record entries) are performed in electronic health records just after the training bout.

**Equipment**

The following equipment is required

- Phone
- Laptop
- Stopwatch
- Visual analog scale measure
- Click counter
- Poster with BORG RPE 20 scale and HIIT intervals (laminated A3)
- Exercise bike

**Attendance and compliance**

Registration of missing attendance, cancellation, or deviation from the described HIIT or resistance training is registered in the participant's training log. If a patient are missing attendance the primary investigator is contacted by email. The participant name, study ID, and the number of the training session are noted in the email.

**Registration of symptoms before training**

Before the beginning of any training bout, the participants are questioned if they have any symptoms which may influence the training (e.g. pain, discomfort, etc.). Following guiding sentence can be used (*italics)*:

*‘Before we begin, do you have anything that may influence your training today? It could be soreness, muscle pain, tenderness?’*

The participant answer is registered as precisely as possible in the training log, and the participant is asked to assess the intensity of the symptoms using a Visual Analog Scale (VAS). One note could, for example, be ‘*Pain in the right knee, all the time, VAS 5’*, or *‘Nausea, periodically, VAS 5’*. The majority of participants will be unfamiliar with the VAS, why an explanatory explanation will be needed. The following guiding sentence can be used (*italics):*

*‘This is a VAS-measurer. It will be used during your training course to access your symptoms if you have any. You adjust the vertical line, according to the severity of your symptoms. The one end represents no symptoms at all and the other end represents the most severe reaction you can think of.’*

On the back of the VAS-measure, the physiotherapist reads the correlating number and register this value in the training log.

The participant is informed before the individual training bout to mark if there is any change in symptoms or if any new arises. The following sentence can be used:

*‘I need you to tell me if during the training today arises any pain, discomfort, or any other symptoms. If you do not tell me anything, I will assume that there is none.’*

**During the training**

Pain, discomfort, or other symptoms during the training are noted in the training log using the participant's full statement and the severity of the symptom by the VAS.

**Introduction to High-Intensity Interval Training (HIIT)**

The HIIT is performed on an exercise bike (Monark) in intervals with ‘active breaks’ between intervals. Four minutes of warm-up is performed, before and after the HIIT intervals on approximate 30% of maximum capacity. This load is used in the ‘active breaks’ between intervals with a duration of 3 minutes (Figure 1.)


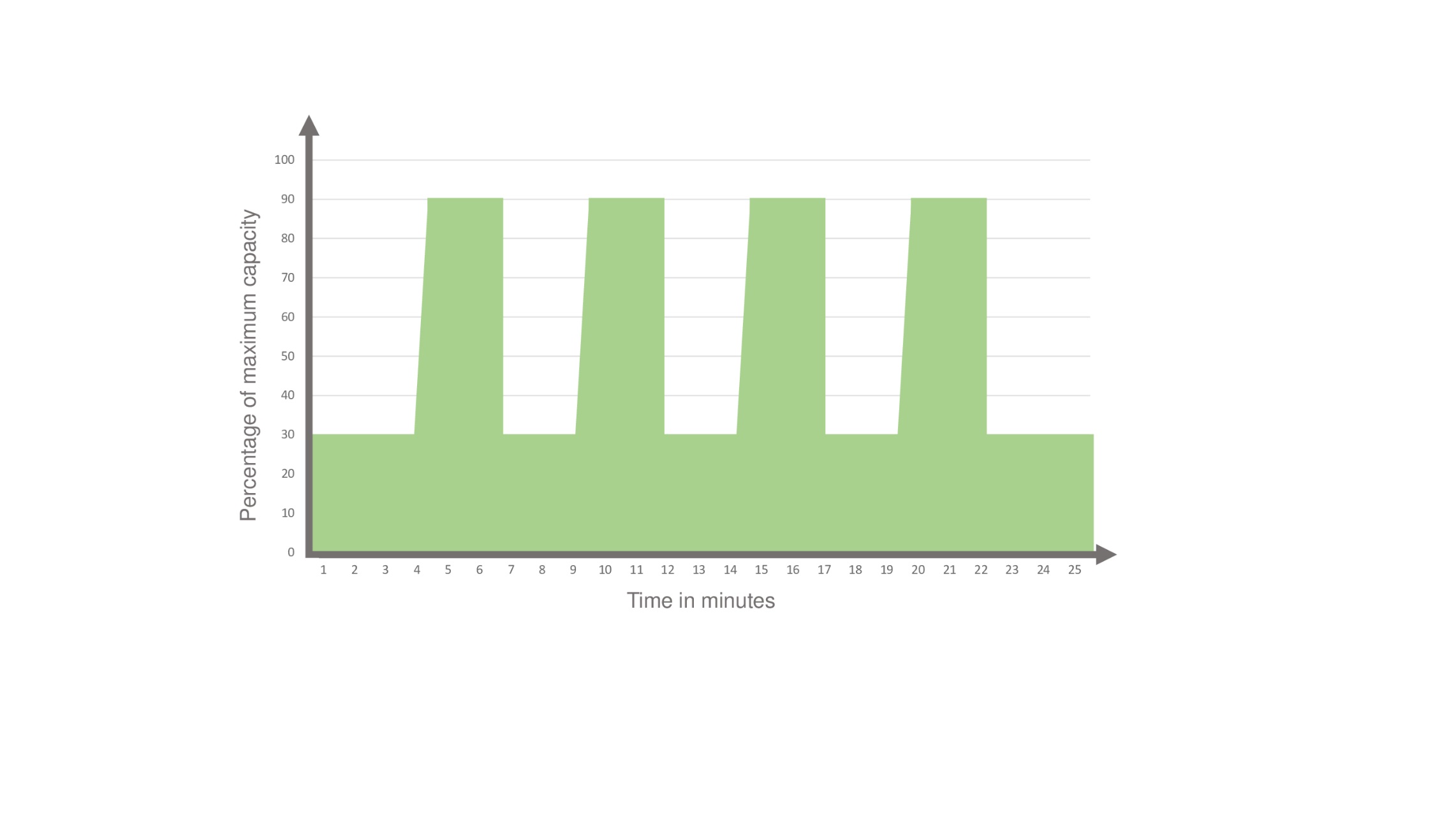


The aim for the participant to go through the four HIIT intervals of each 2-minutes with an intensity of >90 % of maximum heart rate, measured by the CPET at baseline. The maximum heart rate is measured by Polar heart rate monitor (see below) and borg RPE 20 scale.

**Start of training**

The participant is instructed in the content of the HIIT and the ergometer bike is adjusted, so the participant is comfortable with an appropriate flexion/extension of the knee when the pedals are rotated. The height of the seat is noted in the training log for use in future training sessions.

The following instruction is used before the start of HIIT:

‘*The training you are now going to perform consists of high-intensity cardio training followed by resistance training, which together takes 1hour – to 1hour and 15 minutes. The cardio training is performed as interval training on an exercise bike, and the resistance training is performed in machines.*

*Interval training means that you will begin on a fairly low load, but in periods the load will increase to near your maximum capacity, this is then followed by a ‘low load period’. It’s like biking across a small hill. This will be repeated four times – i.e. you are biking across four small hills. When the load increases you will be out of breath, but this will subside when the load is reduced. Every hill takes 2 minutes to come across.*

*You must keep pedaling during the intervals, even if it feels hard and you become exhausted. If the hills are too hard for you to pass, we will adjust the load down, until you succeed. I will be here all the time and tell you what you shall do and keep you motivated. If you become ill during your training, you must tell me.*

The participant is then instructed in usage of the BORG RPE 20 scale (figure 2.)

The following instruction is used:

*‘ During the training, you will be shown this figure several times while you are biking. When I show you the figure, you tell me/point on the number which best describes the exertion you are in. At the right of the table, there are some statements which can help you assess the right number’*

| (0%) | 6 |  |
| --- | --- | --- |
|  | 7 | Extremely light |
|  | 8 |  |
|  | 9 | Very light |
|  | 10 |  |
|  | 11 | Light |
|  | 12 |  |
| 50% | 13 | Somewhat hard |
|  | 14 |  |
|  | 15 | Hard (heavy) |
|  | 16 |  |
|  | 17 | Very hard |
|  | 18 |  |
|  | 19 | Extremely hard |
| 100% | 20 |  |

**Figure 2** Borgs Ratings of Perceived Exertion.


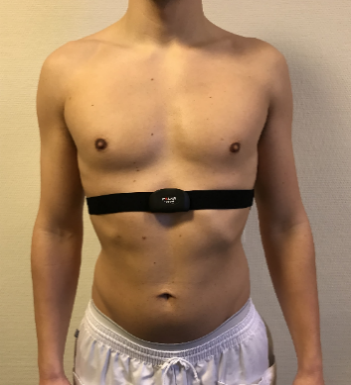
The participant is equipped with a heart rate monitor (Polar) which is placed just below the chest. The part of the heart rate monitor which has contact with the chest is soaked in water before application. The monitor is placed just above the distal end of the sternum (see picture). The heart rate monitor is paired with the corresponding watch which you keep during the training and monitor and register the heart rate at the appropriate times.

**HIIT and progression of load**

The primary parameter to monitor is the heart rate. At each interval begins the stopwatch is used to register how much time is spend above 90 % of maximum heart rate. The value of the 90 % heart rate for each participant is already present in the training log as a result of the baseline testing on CPET.

During the training session, the physiotherapist will give verbal support, especially during the intervals, to enable the participant to continue to pedal with the aimed load.

The following instruction can be used:

*‘In a moment you will bike across a hill. You will experience that the load will increase’*

*‘You are doing well – keep pedaling’*

After each interval, the physiotherapist will ask the participant to assess how hard the interval was using the RPE-scale and register the amount of time spend above 90 % of the maximum heart rate.

After all four intervals are performed, the participant is asked how hard the training in total have been using the Borg RPE scale. If the patient answers below 15 and/or does not reach 90 % of maximum heart rate during the intervals two sessions in a row, the load should be increased by 10 % during the next session. If you observe that the participant does not show the relevant exertion the same applies. Regression is performed by the same principle, or during the HIIT by 10 % of the load.

**After HIIT or discontinuation**

The needed data points are registered in the training log and the heart rate monitor is removed. The bike and monitor are cleaned with an ethanol wet-wipe and the monitor is sent to cleaning.

**Resistance training**

After the HIIT the following three exercises are performed in this chronological order: Chest press, pull down and leg press (pictures 1, 2, and 3 below). The exercises are performed in the TechnoGym machines with Element+. The aim is to perform 3 sets of 10 repetitions with a tempo of 2 seconds of concentric force, followed by 2 seconds of eccentric force. The physiotherapist monitors and guides to correct completion and the repetitions registered with a click counter. Between each set, a break of 1-2 minutes is held. The length of the breaks are monitored by a stopwatch.

1. Chest press 2. Pull down


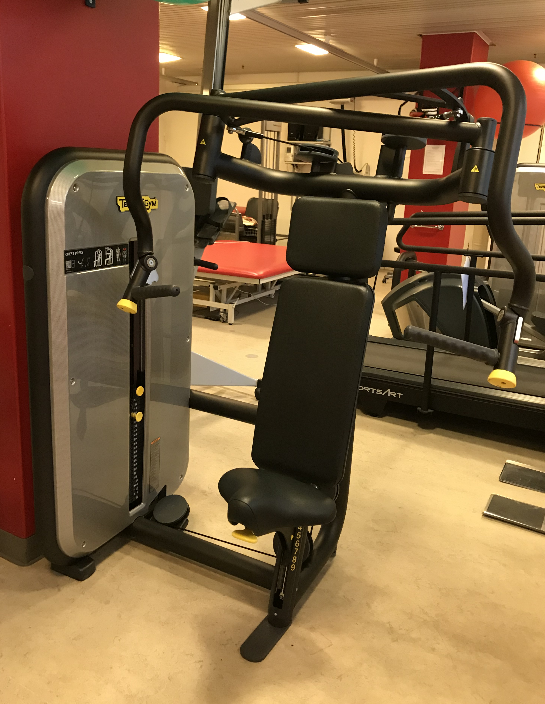

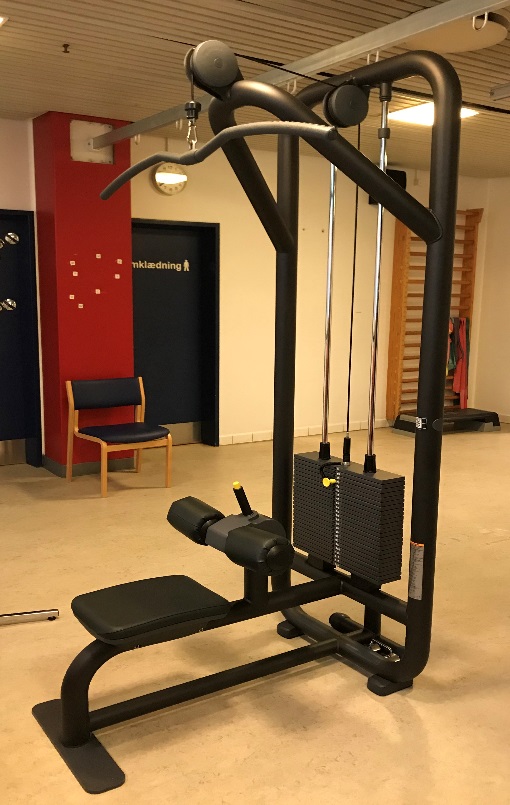


3. Leg press


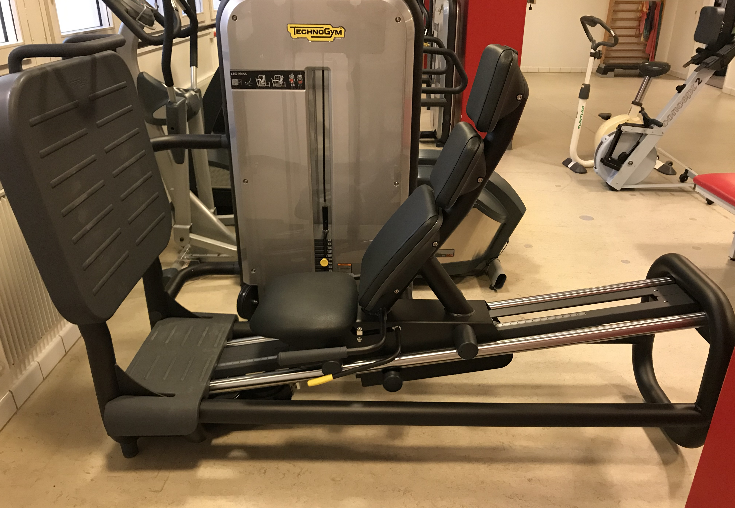


**Execution of resistance training**

During the first training session, the load which will be used for training, is assessed by an indirect 1-RM test. The indirect 1-RM is performed in the above-mentioned machines. Through good clinical judgment, an estimated start load is chosen with the aim of 5-8 repetitions.

If the patient is unable to perform an exercise at baseline, another exercise with the focus on the same major muscle groups is proposed using the physiotherapist's clinical judgment and will replace the exercise in future training sessions. This should be noted in the training log

The participant's future load is calculated by an RM calculator based on Brzyckis formula. E.g. if the participant could perform 8 repetitions of 50 kg, this is will be a 1-RM of 62.1 kg and a training load of 46.6 kg (10 RM). *NOTE: A separate manual of indirect 1-RM on each of the above mentioned have been developed and can be requested by contacting the corresponding author*, *but follows the below instructions of each exercise’*

The training load has the following progression:

Week 1: 65 % of 1-RM

Week 2: 70% of 1-RM

Week 3-5: 75% of 1- RM

Before beginning the resistance training the following instruction are given:

*‘You now have to perform the resistance training. It will be performed in machines with a load, we have estimated from the test you prior have performed. It’s the idea that you perform each exercise three times, with 10 repetitions each time. You will get a short break between sets. I will be there to help you and count the repetitions’*

The exercises are performed in even tempo with 2 seconds of concentric force and 2 seconds eccentric force. The physiotherapist will verbally guide the participant to correct execution.

**Chest-press:** this exercise is performed optimally with the arms horizontally and the hips and knees in 90 degrees flexion. The hands will begin just in front of the shoulder joints and the participant presses the handles forward until the arms are stretched. The participant's hand should not get behind the shoulders and the elbows should not be overextended.


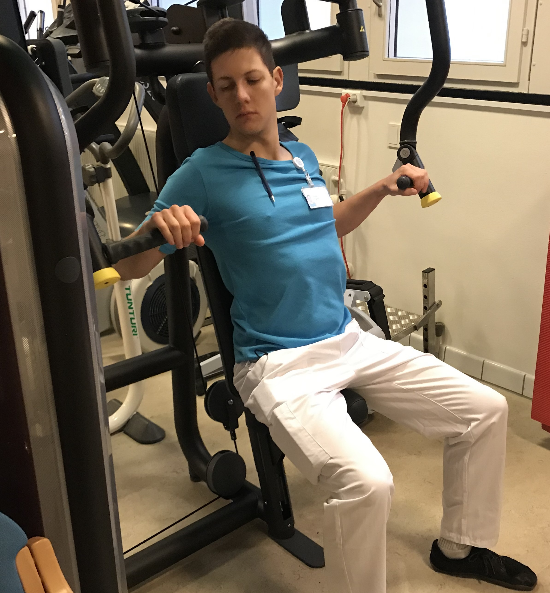

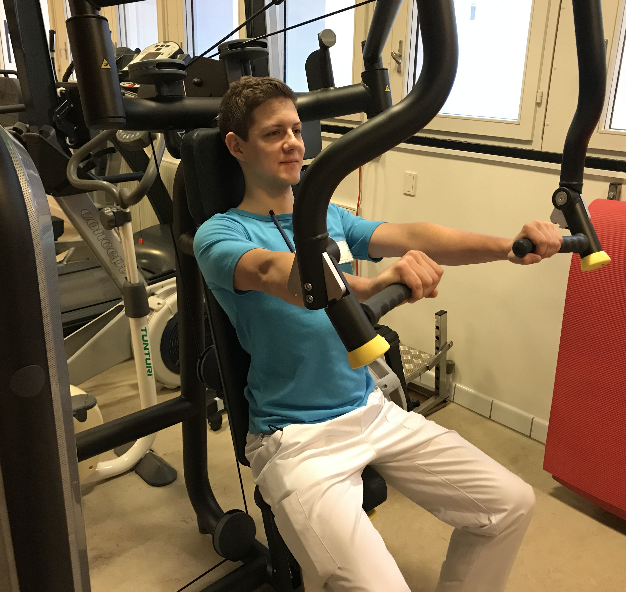


**Pull-down:** The exercise is performed optimally with both the knee and hips in a 90 degrees flexion. The arms begin in a stretched position and the head slightly tilted. The participant will pull the rod down to the chest.


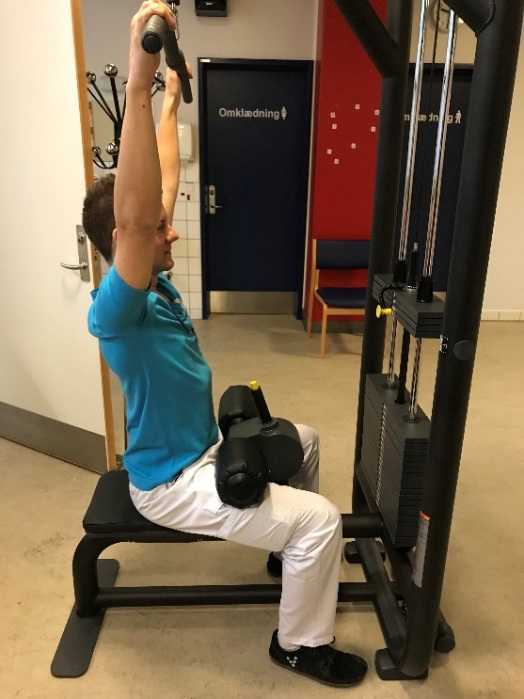

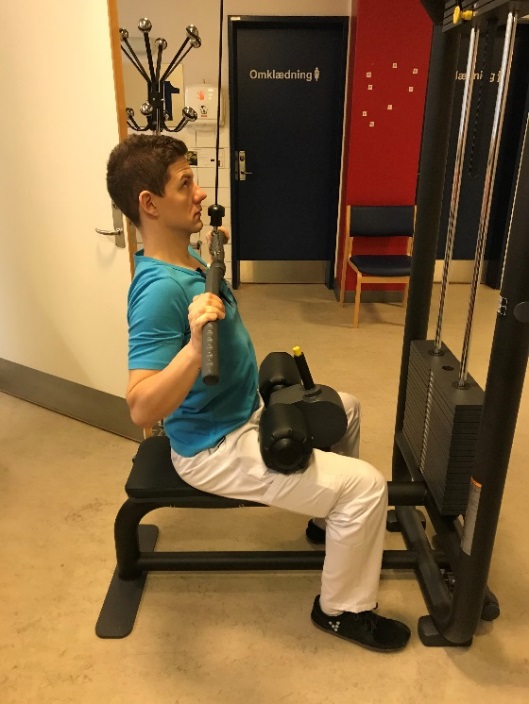


**Leg press:** The exercise is performed optimally with the feet placed vertically on the plate with the toes just below the third groove from the top and with the knees in 90 degrees flexion. The participant then pushes so the seat will move away from the plate, and stops just before the legs become overextended. Then the participant should slowly bend the knees again.


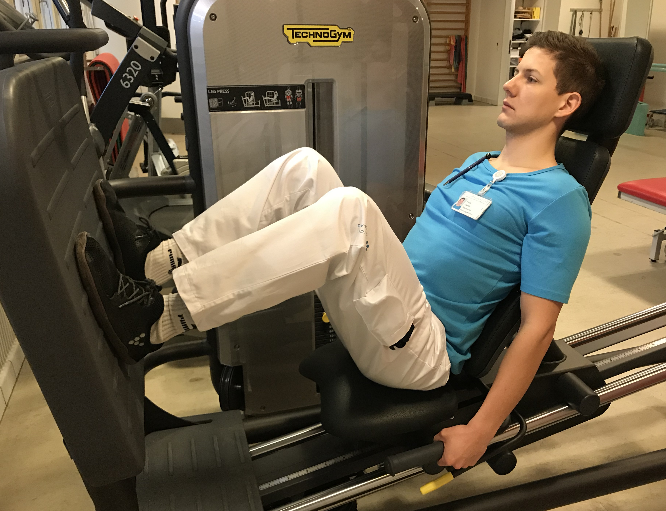

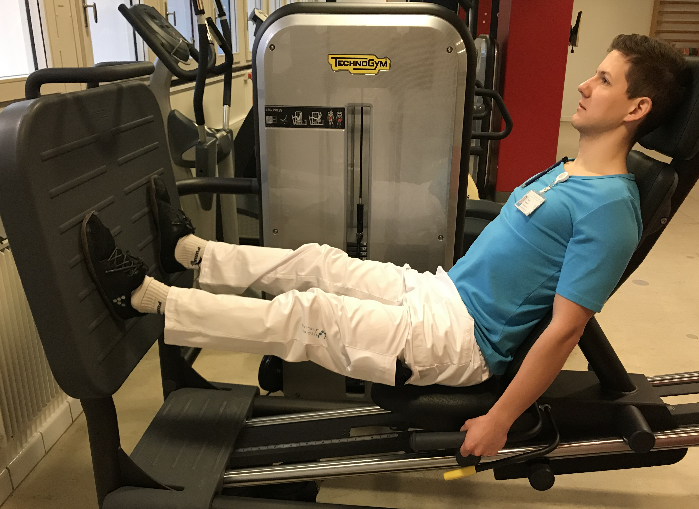


**Adjustments**

The participant's individual settings in each machine are noted in the training log, so these can easily be reapplied later. Amount of repetitions or deviations from protocol are noted in the training log. If pain or discomfort arises these are also noted. If the patient can perform more than 10 repetitions or suggest that the load is too light – the load can be adjusted on an individual basis and this is registered in the training log.

**Encouragement to physical exercise (Home-based)**

As supplementary to the above HIIT and resistance training the participants are encouraged to perform 30 minutes of low to medium intensity exercise each day without training. This could be brisk walking or light biking.

## Appendix 2: Dietician manual – protein supplements

**Translation:**

The dietician manual was translated into English from original Danish. The original protocol contains information about registration in electronic health records and other local information which is omitted in the translated version. Further, the protocol contains information about principles for nutrition that adhere to local protocol and specific Danish conditions, which is also omitted. The original Danish protocol can be requested by contacting Rasmus Dahlin Bojesen [radb@regionsjaelland.dk](mailto:radb@regionsjaelland.dk) or [rasmus.bojesen@gmail.com](mailto:rasmus.bojesen@gmail.com).

**Measurements**

At baseline the following measurements are performed by a medical doctor:

- Patient-Generated Subjective Global Assessment (PG-SGA)
- Weightloss during the last 2 weeks, month, 3 months and 6 months
- Current weight and height
- Skinfold measurements: Triceps, biceps, subscapular, iliac crest, and calf. All on the non-dominant side – in standing position.
- Circumference of the wrist, upper arm, abdomen, and thigh at punctum maximum on the non-dominant side.
- Blood work: Zinc, phosphate, magnesium, vitamin-D, calcium, potassium, sodium, chloride, BUN, creatinine, and albumin.

**Supplements**

All patients are given the following supplements to ingest at home:

- 0.4 g/kg bodyweight protein supplement x 2 daily (Just after exercise and before sleep) using a TMP-90 Shake®. The minimum dosage is 30 g.
- A multivitamin daily (Apovit Multi®)
- 38 µg vitamin-D and 400 mg calcium (Unikalk Mega®)

The above are delivered to the patient at the end of baseline testing along with written, practical, and oral instructions. The TMP shake is tested at the consultation with the medical doctor.

If the patient already takes a multivitamin, d-vitamin, or oral iron, it’s discontinued during the intervention in favor of the study’s supplements.

**Nutritional counseling**

A trained dietician will perform the nutritional counseling, in a 1 hour and 30 minutes consultation, within the first week of intervention. Before the consultation PG-SGA, anthropometric measurements, weight loss, height, and weight are measured, blood work performed and the start of protein supplements begun.

Nutritional counseling consists of:

- 24-hour recall of intake
- Diet anamnesis
- Calculation of current intake of kcal/day
- Calculation of contents (protein, fat, carbohydrates)
- Estimation needed energy requirements by Harris-Benedict equation with factor 1.3-1.5
- Estimation of current protein need with 1.5-1.8 g protein/kg bodyweight
- Nutritional counseling about change in diet content
- Assess if the patient is at risk and should start additional nutritional support

**Qualifications**

The nutritional counseling was performed by a certified dietician with 1.5 years of clinical experience with counseling patients with colorectal cancer.

**Materials**

- VITAKOST application to calculate current intake through diet anamnesis (<https://www.vitakost.dk/da/hjem>)
- Infographics (see below)
- Folders *‘Nutrition to the malnourished patient’* (Danish: Kost til underernærende) and *‘The small appetite’* (Danish: Den lille appetite) – can be requested by the author
- Tables with protein content of different foods – locally adapted
- List of oral supplements (see below)

**24-hour recall and estimation of current intake**

As the first part of the consultation, the dietician will collect data on current energy- and protein intake through a combination of 24-hour recall and diet anamnesis. Further social conditions and the severity of any diet-related symptoms are assessed. The diet anamnesis should give information about the current quality and quantity of food intake, and its components of macronutrients. These data will be directly collected in VITAKOST which will give an estimation of the above nutrient distribution and energy intake. Further, the diet anamnesis should give the dietician knowledge of meal patterns, preferences, allergy and intolerances, cooking methods, and food and diet prejudices.

**Estimation of current need**

The basic energy consumption is calculated using the Harris-Bennedict formula and is multiplied by the activity/stress factor of 1.3-1.5. For most participants in which weight gain is favorable, it will be 1.5.

Protein consumption is aimed at 1.5-1.8 g protein/kg bodyweight

**Counseling**

Together with the patient, the dietician develops a plan to meet the goal. This will be developed during the consultation using the infographics and through educational principles which focusses on empowerment. The plan will result in specific written advice that along with folders and infographics will be given to the participant.

There are no specific limitations or demands for the energy distribution between macronutrients. However, a good rule of thumb is 25-30 gram protein per meal. There are no specific demands to the type of fat ingested, but it should be noted that the participants should receive the recommended amount of essential fatty acids. Further, any recommendation of extra intake of fat should be in monounsaturated fatty acids, if the patient is malnourished, diabetic, or have a history with high lipids in the bloodwork. These patients should receive extra education in fat quality and quantity.

**Oral nutritional support**

For patients with risk factors of malnutrition (e.g. low BMI, current weight loss, a decline in food intake, High PG-SGA score) oral nutritional support (ONS) will be discussed, using the table (see below) and by demonstration. Both prescription and non-prescription ONS will be offered according to preference and severity of malnutrition or risk. The ONS should be taken outside meals and should not replace the protein supplements already given.

If a prescription is needed it will be performed by a medical doctor

**Adherence**

**F**ollow up is not performed on nutritional counseling or dietician advice. At the preoperative testing, the remainder protein supplement is collected and weighed, and the vitamin supplements counted to estimate the percentage of ingestion. The percentage is calculated as the following:

Percentage = 100 * (Protein ingested / (0.8 g/kg bodyweight*preoperative days))

The aim of the protein supplement is >65 %.

A Danish version of infographics used can be found at <https://ernaeringsfokus.dk/-/media/ernaeringsfokus/materialer/2018/protein-in-food--da--1-6-2.jpg>

**List of oral supplements**

| **Product** |  | **Content per unit** | | **Tastes** |
| --- | --- | --- | --- | --- |
|  |  | **Energy, kJ** | **Protein, gram** |  |
| **Prescription medical oral supplements** | | | | |
| **Fresubin 2 kcal Drink**  **200 ml**  Fresenius K. | 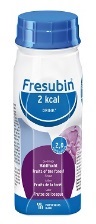 | 1680 | 20 | Peach, Mixed berry’s, Lemon, Vanilla, Neutral, Caramel, Cappuccino, Chocolate |
| **Nutridrink Yoghurt Style**  **200 ml**  Nutricia | 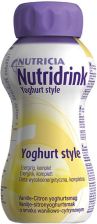 | 1260 | 11,8 | Raspberry, Vanilla/Lemon, Peach/orange  (Low lactose) |
| **Nutridrink Compact**  **125 ml**  Nutricia | 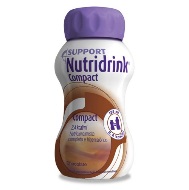 | 1253 | 19,2 | Strawberry, Banana, Mocca, Apricot, Chocolate |
| **Nutridrink Protein**  **200 ml**  Nutricia | 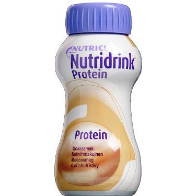 | 1260 | 34 | Mocca, Apricot, Chocolate, Berry’s, Vanilla |
| **Nutridrink Juice Style**  **200 ml**  Nutricia | 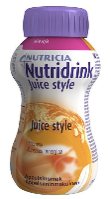 | 1270 | 7,8 | Strawberry, Orange, Apple;  Black currant, Mixed Berry’s |
| **Non-prescription, oral supplements** | | | | |
| **Arla Protino**  **125 ml/1 bottle**  Protein supplement Arla | 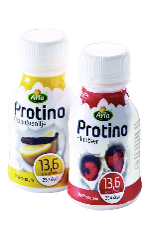 | 1062 | 13,6 | Vanilla/Lemon, Raspberry, blueberry |
| **Arla Protin**  **1 unit / 175 ml**  Protein supplement, Arla | 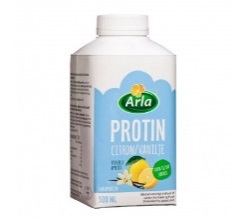 | 1006 | 10,6 | Vanilla/Lemon, Raspberry, peach/passionfruit, orange. |
| **Protino Dessert**  **80 gram/1 Cup**  Arla |  | 447 | 5,8 | Appel/Cinnamon, Rhubarb/vanilla |

## Appendix 3: Intravenous iron treatment and correction of other medical factors

Intravenous iron treatment

**Clinical indication**

Preoperative hemoglobin ≤ 7.0 mmol/L (11.3 g/dL) for both men and women.

**Exclusion criteria**

- P-Ferritin > 800 µg/L
- Chronic kidney disease with eGFR < 30 ml/min/1,73 m^2^
- Contraindications: Hematological diseases, active serious infection, Porphyria, hemochromatosis, liver cirrhosis, or active hepatitis.
- Known allergies for iv. Iron

**Drug and dosage:**

Iron(III)-isomaltoside 1000 (MonoFer®, Pharmacosmos, Denmark), 100 mg/ml

Total dosage was estimated individually using the table below

| Hemoglobin (mmol/L) | Bodyweight (kg) | |
| --- | --- | --- |
|  | 50-70 kg | > 70 kg |
| < 6.2 (mmol/L) | 1.500 mg | 2.000 mg |
| ≥ 6.2 (mmol/L) | 1.000 mg | 1.500 mg |

For a patient with bodyweight below 50 kg individual dosage is calculated by Ganzoni´s formula:

Total dosage (mg Fe) = Bodyweight* (kg) x (normal hemoglobin (mmol/L) – current hemoglobin (mmol/L)) x 3,84 + iron storage (500 mg).

*Bodyweight is calculated from an ideal weight on a BMI of 25.

Maximum dosage per infusion is 20 mg iron(III)-isomaltoside/kg bodyweight. If the total dosage exceeds the maximum dosage per infusion, the remainder dosage is given 1 week after the first infusion.

**Practical’s**

Treatment with iron(III)-isomaltoside is administered by the department of surgery at the surgical ward on the same day of the outpatient visit. Before infusion, vital parameters are measured and recorded. Iron(III)-isomaltoside 100 mg/ml mixed with 100-500 ml isotonic NaCl-infusion fluid is administered over 30 minutes for ≤1000 mg and 45-60 minutes with a dosage ≥1000 mg. After end infusion, the patient stays for observation at the department for a minimum of 30 minutes for adverse reactions. If the patient has no symptoms of adverse reactions and vital parameters are stable the patient can subsequently be discharged. Patients are informed of the possibility of delayed reactions and to contact the department if they occur before discharge.

Medical optimization

As part of the baseline test, a full medical checkup is performed by the principal investigator. Current symptoms, previous medical history, mental wellbeing, and physical function are assessed. One hour is estimated per patient.

Expanded bloodwork is performed including Leucocytes, CRP, Hemoglobin, thrombocytes, albumin, creatinine, BUN, INR, MCV, MCHC, folate, B12, vitamin D (1, and 25), serum iron, transferrin, transferrin saturation, ferritin, Hba1c, zinc, phosphate, potassium, sodium, and chloride.

**Referrals**

If during the medical checkup, expanded bloodwork or CPET unknown medical conditions are discovered or suspected, a referral to the relevant clinical department will be performed.

**Medication**

If the patient receives more than three different kinds of medications, full scrutiny of the medications are performed in regards to the guidelines of the Danish Medical Authority (Sundhedstyrelsen). Discontinuation of drugs is considered based on the current Danish guidelines: <https://www.sst.dk/da/viden/laegemidler/medicingennemgang/seponeringslisten>.

**Follow-up**

Patients are weekly contacted and questioned on their well being both somatically and mentally. Any progression of symptoms, lack of adherence to the protocol is discussed with the principal investigator, and the responsible surgeon.

## Appendix 4: Reporting of exercise intervention in regards to CERT guidelines

**Item 1: Detailed description of the type of exercise equipment**

The following equipment was used for the intervention:

- Monark 927 E Cardio Care Ergometer bike
- Polar A300 Heart rate monitor
- Chess press (TechnoGym)
- Pull down (TechnoGym)
- Leg press (TechnoGym)
- Laminated A3 Board with intervals and Borg RPE-6-20 scale
- Click counter
- Stopwatch
- 1 kg extra weights for TechnoGym machines

**Item 2: Detailed description of the qualifications, expertise and/or training**

All training was performed by certified physiotherapists with varying degrees of clinical experience. All physiotherapists had 8 hours of education in the protocol before initiation and had to perform a training session on a colleague and themselves.

**Item 3: Description of whether exercises are performed individually or in a group**

All training sessions were performed individually.

**Item 4: Description whether exercises are supervised or unsupervised; how they are** **delivered**

The training was performed as supervised training in an in-hospital setting at the department of physiotherapy. Two separate rooms were used: one for High-intensity interval training and one for resistance training. Simultaneously training of other patients in the room was not performed.

The home-based exercise component was unsupervised and not monitored.

**Item 5: Detailed description of how adherence to exercise is measured and reported**

Adherence was measured on two levels. It was measured as the percentage of training sessions held out of the maximum possible. This included cancellation from the patient, the department of physiotherapy, and due to other hospital engagements such as colonoscopies or referrals to other departments. Further, it was measured as the percentage training sessions which reached the aim of a minimum of 4 minutes of heart rate above 90 % of maximum heart rate determined by the cardiopulmonary exercise test at baseline.

**Item 6: Detailed description of motivation strategies**

The patients had a motivational interview as part of the baseline testing with a medical doctor outlining the importance of each part of the multimodal intervention, including the supervised training and the home-based exercise. The motivational interview was not structured. The medical doctor contacted the patient each week by phone or by showing up before a training session to discuss the progression of the intervention. This was also not structured and no time limit was predefined.

At each training session, the participant was informed by standardized phrases (see appendix 1) about the training and the expectations of the physiotherapist. No formal motivational strategy was predefined of motivation during the exercise, but all physiotherapists were encouraged to express vocal support during the high-intensity intervals.

**Item 7a: Detailed description of the decision rule(s) for determining exercise progression and Item 7b: Detailed description of how the exercise program was progressed**

High-intensity interval training: If the participant had a total Borg RPE 6-20 scale below 15 and/or does not reach 90 % of maximum heart rate during the intervals two sessions in a row, the load would be increased by 10 % during the next session. The same applied if the physiotherapist observed that the participant did not show the relevant exertion the same applies. Regression is performed by the same principle, or during the HIIT by 10 % of the load.

Resistance training: The training load had the following aimed load progression: Week 1: minimum of 65 % of 1-RM, Week 2: minimum of 70% of 1-RM, Week 3-5: minimum 75% of 1- RM. No regression or progression manual was defined outside the above.

**Item 8: Detailed description of each exercise to enable replication**

Each exercise is described fully in appendix 1: *Exercise manual*.

**Item 9: Detailed description of the home program component**

The participants were encouraged to stay active with a minimum of 30 minutes of medium to low-intensity exercise, on the day´s which no supervised training was performed. Medium intensity exercise was described verbally (e.g. Brisk walking, light biking) by a medical doctor as part of the motivational interview, and by the physiotherapist when concluding each training session. Adherence to the home program was not monitored.

**Item 10: Describe whether there are any non-exercise components**

The intervention was multimodal, with concomitant nutritional- and medical optimization. This is described elsewhere.

**Item 11: Describe the type and number of adverse events that occur during exercise**

No specific training adverse events happened during the exercise. In total three adverse events happened in the intervention period: One patient developed a urinary tract infection, one had changed on their ECG on baseline testing, but without the need for treatment or further diagnostics, and one patient developed an abscess of the tumor. None of the adverse events was judged causal to the intervention.

**Item 12: Describe the setting in which the exercises are performed**

All training sessions were performed in a hospital setting at the department of physiotherapy. The training was planned to be performed within normal office hours Monday, Wednesday, and Friday. It was aimed to be performed by the same trainer each time and at the approximately same time of day, but was not always possible. All trainers had undergone seven hours of education in performing the training session before training participants on their own. No training was performed on holidays or weekends. If the physiotherapy department was closed, the patient had other engagements at the hospital which could not be postponed e.g. colonoscopy, or the patient canceled, the training session was not performed and was not replaced by another training session. When planning the intervention at baseline testing a minimum of 10 training sessions had to be possible between, but not including baseline and preoperative testing.

**Item 13: Detailed description of the exercise intervention**

The exercise intervention consisted of in-hospital training of 1hr and 15 minutes sessions three times a week for a minimum of four weeks. The training consisted of High intensive interval training (HIIT) followed by resistance training. The HIIT was performed as 4x4 with 2 minutes of load aiming at 90 % of maximum capacity.in the intervals with an ‘active break’ on 30 % of maximum capacity between lasting 3 minutes. Before the first interval and after the last interval a warm-up/down on 30 % of maximum capacity was performed for 4 minutes. The resistance training consisted of three exercises in machines: Chest press, pull down, and leg press each aimed at three sets of 10 repetitions. If the participant was unable to perform the exercise at baseline, it was switched to another exercise focusing on the same major muscle groups using the physiotherapist's expertise. All resistance training was performed using 2 seconds of concentric force followed by 2 seconds of eccentric force. All sets had a break between 1-2 minutes.

Further details can be found in appendix 1.

**Item 14a: Describe whether the exercises are generic (one size fits all) or tailored**

The exercise was tailored to the individual, but within a generic framework consisting of High-intensity interval training on a bike, followed by three resistance training exercises focusing on the major muscle groups.

**Item 14b: Detailed description of how exercises are tailored to the individual**

High-intensity interval training: The only load was tailored as described below (item 15).

Resistance training: If the participant was unable to perform the exercise in a machine. The exercise was replaced by another focusing on the same muscle groups using the physiotherapist's expertise.

**Item 15: Describe the decision rule for determining the starting level**

The starting level for the HIIT was estimated using Cardiopulmonary Exercise Testing using a step ramp on a Vyntus CPX Jaeger system. The maximum heart rate was recorded and used for monitoring of the HIIT training. The wattage at maximum oxygen uptake was used as guidance for load estimation.

Resistance training: Indirect 1-RM measurements were performed at the first training session using Brzyckis formula, and used for estimation of load during the training.

**Item 16a: Describe how adherence or fidelity is assessed/measured and Item 16b: Describe the extent to which the intervention was delivered as planned**

Of the 94 possible training sessions 82 (87 %) were performed, but with great variability between patients (54 % to 100 %). The mean Borg’s scale estimation for training sessions was 17 (R: 15 -18.5). In three patients we were not able to get an accurate heart rate reading either due to severe scoliosis (n=1) or atrial fibrillation (n=2). For the monitored training sessions, 87 % met the goal of 4 min > 90 % heart rate. For several patients we observed no drops in heart rate between bouts in the low-intensity phase, thus 40 % of training sessions were performed with more than 8 minutes of > 90 % in heart rate during the high interval bouts.

Fidelity was not measured or assessed
